# Current rates of *Helicobacter pylori* infection and antibiotic resistance in the eastern coast of China: a single center study

**DOI:** 10.3389/fcimb.2025.1561778

**Published:** 2025-04-01

**Authors:** Hui-Jin Chen, Yu-Ting Si, Luan Luan, Jin-Xin Lai, Ji-Liang Wang, Yu-Rong Tang, Liang Wang

**Affiliations:** ^1^ Department of Laboratory Medicine, Shengli Oilfield Central Hospital, Dongying, Shandong, China; ^2^ Medical Technology School, Xuzhou Medical University, Xuzhou, Jiangsu, China; ^3^ Guangdong Provincial People’s Hospital (Guangdong Academy of Medical Sciences), Southern Medical University, Guangzhou, Guangdong, China; ^4^ Intelligent Medical Engineering, School of Medical Informatics and Engineering, Xuzhou Medical University, Xuzhou, Jiangsu, China

**Keywords:** prevalence, antibiotic resistance, string test, H. pylori, qPCR

## Abstract

**Background:**

*Helicobacter pylori* (*H. pylori*), linked to gastric cancer, lacks regional data on infection rates, resistance, and risks in Dongying, China.

**Methods:**

A pilot study (N = 314) using non-invasive string tests and qPCR assessed *H. pylori* prevalence and antibiotic resistance. Logistic regression identified risk factors from survey data.

**Results:**

The *H. pylori* infection rate in Dongying was 24.84% (78/314, 95% CI: 20.06%-29.62%). Of the infected individuals, 60.25% (47/78, 95% CI: 49.40%-71.12%) and 46.15% (36/78, 95% CI: 35.09%-57.22%) showed resistance to clarithromycin and levofloxacin, respectively.

**Conclusions:**

This pilot study provides preliminary data with valuable insights for preventing the transmission of *H. pylori* infection among the urban population in the East Coast region of China and offers guidance for the personalized treatment of drug-resistant *H. pylori* infections.

## Graphical Abstract

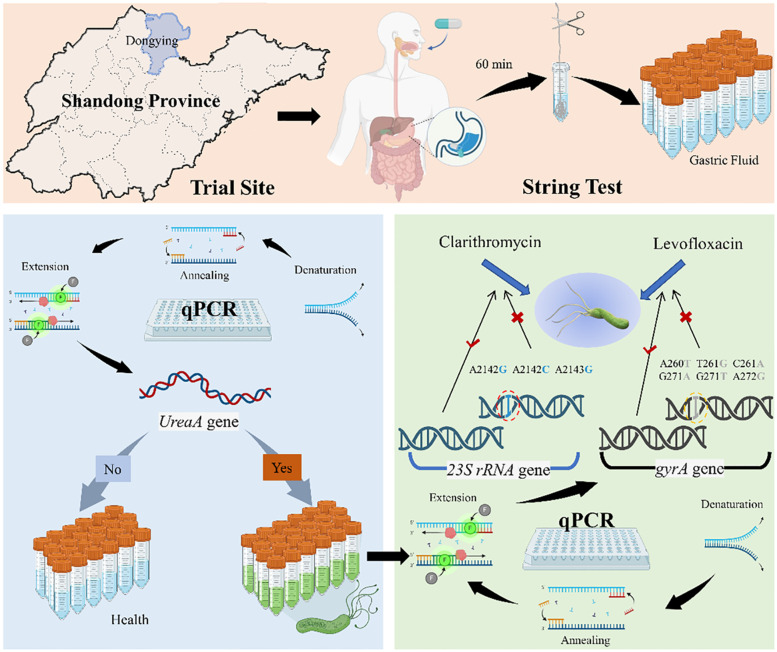

## Introduction

In 1983, Marshall and J. Robin Warren successfully cultured and identified *Helicobacter pylori* (*H. pylori*) in gastric mucosa for the first time (Yamada et al., 1994). *H. pylori* is a Gram-negative, microaerophilic bacterium known for its ability to colonize the human stomach persistently. By producing urease, *H. pylori* can neutralize gastric acid, allowing it to adapt to the acidic environment of the stomach—a key feature of its pathogenic mechanism. Moreover, *H. pylori* uses a variety of virulence factors to target host cellular proteins. These factors finely regulate inflammatory responses and contribute to diverse damage to the gastric mucosa. *H. pylori* infection has been conclusively linked to the development of chronic gastritis, peptic ulcers, gastric cancer, and MALT lymphoma (Burucoa and Axon, 2017). The International Agency for Research on Cancer (IARC) has classified *H. pylori* as a Group I carcinogen (Yan et al., 2024). Eradicating *H. pylori* can significantly reduce the risk of gastric cancer, and many countries and regions have achieved lower incidence rates of gastric cancer by eradicating this bacterium (Hooi et al., 2017; Chiang et al., 2021). The prevalence of *H. pylori* infection is influenced by various factors such as geographical location, sanitary conditions, and socioeconomic status (Li et al., 2023). A multicenter study conducted by Wang et al. in 2024 found that the prevalence of *H. pylori* infection in the urban areas of China was 27.08%, with significant differences in infection rates across different regions (Wang et al., 2024).

The global issue of antibiotic resistance is becoming increasingly severe, posing significant challenges to the eradication of *H. pylori* (Hong et al., 2024). According to recent studies, the resistance rates of urban residents in China to clarithromycin and levofloxacin are as high as 50.83% and 47.17%, respectively (Wang et al., 2024). The resistance of *H. pylori* to these two antibiotics is mainly attributed to point mutations in key genes, which alter the drug targets and significantly reduce the effectiveness of the antibiotics. Clarithromycin resistance is closely associated with point mutations in the *23S rRNA* gene (*A2142G*, *A2143G*, and *A2142C*), which prevent clarithromycin from binding to the bacterial ribosome’s 50S subunit, thereby blocking its ability to inhibit protein synthesis. On the other hand, levofloxacin resistance is typically related to mutations in the *gyrA* gene (such as N87K and D91G). The *gyrA* gene encodes the A subunit of DNA gyrase, and these mutations decrease the affinity of levofloxacin for DNA gyrase, hindering its ability to interfere with DNA replication. In some cases, overexpression of efflux pumps or changes in membrane permeability may further exacerbate resistance to levofloxacin. These molecular mechanisms significantly affect the therapeutic efficacy of clarithromycin and levofloxacin, influencing clinical treatment strategies and the rate of treatment failure (Greenberg et al., 2011; Molina–Infante et al., 2013). Therefore, detecting resistance gene mutations is crucial for personalized therapy. However, antibiotic resistance develops rapidly due to the widespread use of antibiotics like clarithromycin in the population and the high adaptability of *H. pylori*, making the treatment of *H. pylori* infections increasingly difficult (Thung et al., 2016). As a result, effectively controlling antibiotic resistance and successfully eradicating *H. pylori* has become an urgent public health issue.

When detecting *H. pylori* infection, understanding the patient’s antibiotic resistance is crucial for developing an effective treatment plan. Existing methods for detecting *H. pylori* are mainly divided into invasive and non-invasive categories (Mégraud and Lehours, 2007). Culture and molecular diagnostic methods can provide information on *H. pylori* infection and its antibiotic resistance. Gastric fluid is a sample that can provide real-time insights into the gastric environment. In most current studies, gastric fluid samples are obtained via gastroscopy. However, the invasiveness of this method makes gastric fluid more difficult to collect compared to other body fluids. Previous studies have reported a method for collecting gastric fluid samples using the string test (Domínguez-Bello et al., 2001; Wang et al., 2003, 2023). Introduced in 1970, the string test is a minimally invasive method in which a patient swallows a capsule containing a string, which is then retrieved to collect gastric fluid samples (Beal et al., 1970). Han et al (Han et al., 2023). conducted a single-center study combining the string test with qPCR to guiding *H. pylori* antibiotic sensitive treatment for *H. pylori*. Guided by the results of antibiotic resistance testing, patients receiving 14-day bismuth quadruple therapy achieved an *H. pylori* eradication rate of *H. pylori*. This further confirmed that the minimally invasive string test combined with qPCR is reliable for collecting gastric fluid samples containing *H. pylori* to diagnose infections and antibiotic resistance. This method reduces the need for invasive procedures (such as gastroscopy), makes large-scale screening feasible, and allows for the real-time acquisition of accurate information regarding infection status and antibiotic resistance.

As a burgeoning immigrant city, Dongying has maintained a steady population growth. In 1950, the population of Dongying was 21,504. The population of Dongying is expected to reach 1,410,790 by 2024. With the development of the petroleum industry, Dongying has gradually evolved from a small fishing village into an important industrial city. The first generation of oil workers came from all over the country, exhibiting different dietary habits and lifestyles. To the best of our knowledge, no detailed reports have been published regarding the prevalence, antibiotic resistance, and risk factors of *H. pylori* infection in Dongying. Therefore, it is essential to conduct screening and management of *H. pylori* infection in Dongying, Shandong, which may not only help reduce the infection rate but also effectively prevent the occurrence of serious diseases such as gastric cancer. In this study, we randomly recruited individuals aged 18 to 60 years from Dongying City, Shandong Province, and non-invasively collected gastric fluid samples from participants using the string test method. A combination of string tests with qPCR detected *H. pylori* infection status and antibiotic resistance profiles in the participating population. Additionally, we conducted univariate and multivariate logistic regression analyses based on the collected questionnaire data to identify risk factors associated with *H. pylori* infection. In conclusion, this study has the potential to help optimize prevention and eradication strategies for *H. pylori* infection and further improve public health outcomes in local areas.

## Materials and methods

### Study design

From May 8 to May 23, 2023, we conducted a cross-sectional survey to investigate the prevalence of Helicobacter pylori infection in Dongying City, Shandong Province, China. The survey was conducted through online promotion, inviting urban residents aged 18 to 60 to participate randomly and confirm their participation by providing their information. The following groups were excluded from the study: (1) those who had taken antibiotics, bismuth agents, or antibacterial traditional Chinese medicines within one month, or proton pump inhibitors, H_2_ receptor antagonists, or other drugs within two weeks; (2) those who had undergone *H. pylori* eradication therapy in the past three months; (3) pregnant and breastfeeding women; and (4) those with severe heart, liver, or kidney dysfunction, severe neuropathy, or mental illness. The study adhered to the ethical principles of the Declaration of Helsinki. All participants signed informed consent forms, and the study was approved by the Ethics Review Committee of Guangdong Provincial People’s Hospital (Ethics Approval No. KY-Q-2022-384-02).

### Study data

Under the guidance of the on-site staff, each participant completed the questionnaire. The questionnaire covered the following aspects: (1) demographic characteristics, including sex, age (divided into <30, 30-39, 40-49, and 50-60 years), height, weight, marital status (unmarried and married), and education level (categorized as lower secondary education and below, Intermediate: high school or vocational training, and higher education); (2) lifestyle, including dietary habits, smoking, alcohol consumption, tea drinking, and sharing cups. BMI is defined as weight (kg) divided by the square of height (m^2^) and is used to reflect the obesity status. Based on WHO criteria, underweight was identified as BMI<18.5 kg/m^2^, normal weight as BMI 18.5-24.9 kg/m^2^, overweight as BMI 25.0-29.9 kg/m^2^, and obese as BMI≥30.0 kg/m^2^ (Consultation, 2000).

### Non-invasive collection of gastric fluid via string test

**Figure 1:**
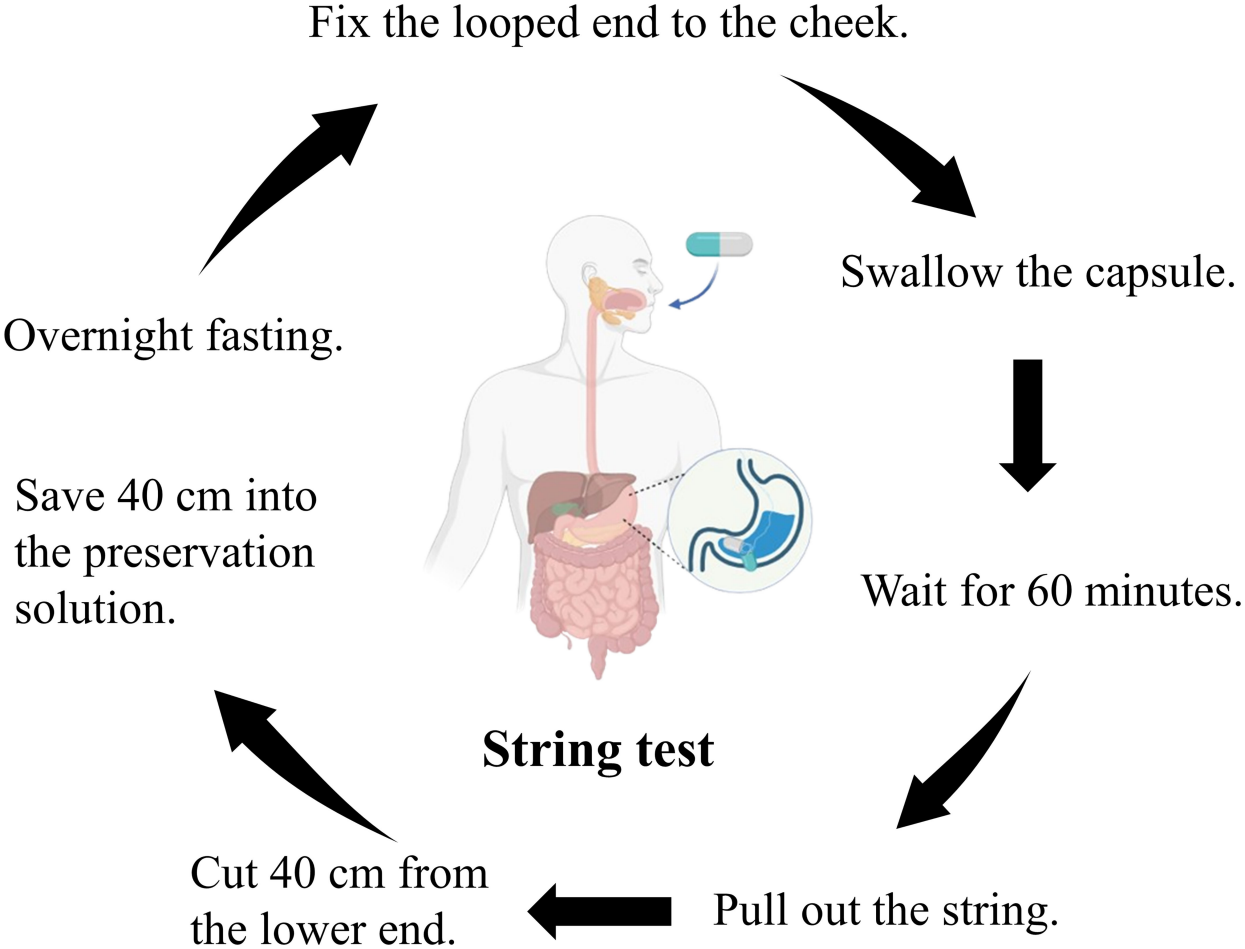
The flowchart of the string test gastric fluid sample collection procedure.

After overnight fasting, gastric fluid was collected using a disposable string test kit (Hongmed-Infagen, Shenzhen, China). The kit included a weighted capsule with an absorbent cotton line. Participants swallowed the capsule, and after 60 minutes, the cotton line, saturated with gastric fluid, was retrieved and cut with sterile scissors. The distal end, which was 40 cm from the cut, was then transferred to a commercial preservative solution (Hongmed-Infagen, Shenzhen, China) (**Figure 1**). The collected gastric fluid samples were sent to the clinical laboratory at the Department of Laboratory Medicine, Guangdong Provincial People’s Hospital, for qPCR analysis.

### Measurement of *H. pylori* infection and antibiotic resistance

DNA was extracted using a nucleic acid extraction kit (Daan Gene, Guangzhou, China) with a Stream SP96 Automated Extractor. The *H. pylori* nucleic acid detection kit (PCR-fluorescent probe method), *H. pylori*, *23S rRNA*, and *gyrA* gene mutation detection kit (fluorescent PCR method) were provided by Shenzhen Hongmei Diagnostic Technology Co., Ltd. DNA (5 µL of DNA and PCR reaction mixture (20 µL) were added to the corresponding wells of an eight-tube strip. PCR conditions included 42°C for 2 min, 95°C for 2 min, and 40 cycles of 95°C for 10 s and 58°C for 45 s. A cycle threshold value ≤35 for *ureA* indicated *H. pylori* infection, and ≤30 for *23S rRNA* and *gyrA* indicated resistance to clarithromycin and levofloxacin.

### Statistical analysis

We used the chi-square test to analyze *H. pylori* infection and compare variables such as gender, age, BMI, education level, and marital status. Sample size calculation was based on the observed infection rate (24.84%), a margin of error of 5%, and a confidence level of 95%. We calculated 95% confidence intervals for all statistical measures, including infection and antibiotic resistance rates. Based on univariate logistic regression analysis, we included factors with a P-value less than 0.5 and those identified in previous studies (Zhou et al., 2023) as significantly associated with *H. pylori* infection in the multivariate logistic regression analysis. Although no factors with a P-value less than 0.5 were found in the univariate analysis, considering their relevance in prior research, we included gender, age, education level, and marital status in the multivariate analysis to further assess their relationship with *H. pylori* infection. Both univariate and multivariate logistic regression were used to identify and confirm independent risk factors, with odds ratios (OR) and 95% confidence intervals (CI) reflecting their impact (P < 0.05). All analyses were performed using R software (version 4.4.1).

## Results

### Basic information about the participants

We randomly enrolled 314 participants for gastric fluid sampling via community recruitment. The average age of the participants was 44.83 ± 10.55 years, with 41.72% (n = 131) being male and 58.28% (n = 183) female. Of the participants, 56.36% had a normal weight, while over 40% were overweight or obese. More than half of the participants had a higher education level, with 62.42% having a higher education degree. There were 272 married individuals, accounting for 86.62% of the total population (**Table 1**).

**Table 1:** Characteristics of the study population.

Categories	Total Number of individual (n=314) (%)	Infected individual (n=78)	Uninfected individual (n=236)	Individual infection rate (%)	*P-*value
Gender					
Male	131 (41.72)	28	103	21.37	0.229
Female	183 (58.28)	50	133	27.32	
Age					
<30	30 (9.55)	6	24	20.00	0.681
30-39	70 (22.29)	20	50	28.57	
40-49	85 (27.07)	23	62	27.06	
50-60	129 (41.08)	29	100	22.48	
Education					
Lower secondary education and below	47 (14.97)	14	33	29.7	0.663
Intermediate: high school or vocational training	71 (22.61)	18	54	25.35	
Higher education	196 (62.42)	46	150	23.47	
Marital status					
Unmarried	42 (13.38)	10	32	23.81	0.868
Married	272 (86.62)	68	204	25.00	

### *H. pylori* prevalence and antibiotic resistance in the Dongying City

The survey showed that the prevalence of *H. pylori* infection in the urban area of Dongying City was 24.84% (78/314, 95% CI: 20.06%-29.62%). The resistance rates of *H. pylori* to clarithromycin and levofloxacin were 60.25% (47/78, 95% CI: 49.40%-71.12%) and 46.15% (36/78, 95% CI: 35.09%-57.22%), respectively (**Table 2**). These data indicate that *H. pylori* infection is relatively common in this region and that there is a significant problem with drug resistance, particularly with notable resistance to clarithromycin.

**Table 2:** The prevalence and antibiotic resistance rates of *H. pylori* in Dongying City.

	Positive, n	Total, n	Positive rate (%)	95% CI (%)
*H. pylori*	78	314	24.84	20.06-29.62
Clarithromycin	47	78	60.25	49.40-71.12
Levofloxacin	36	78	46.15	35.09-57.22

### Risk factors associated with the prevalence of *H. pylori* in the Dongying cohort

Risk factors associated with *H. pylori* infection in the Dongying cohort are shown in **Table 3**. In this cohort, there was no significant difference in the infection rates between males (21.37%) and females (27.32%) (*P* = 0.23). Among the different age groups, the infection rate was highest in the 30–39 age group (OR 1.60, 95% CI 0.59-4.84, *P* = 0.37), followed by the 40–49 age group (OR 1.48, 95% CI 0.56–4.41, *P* = 0.45). Compared to individuals with normal weight (22.60%, 40/177), those who were overweight had a higher infection rate (OR 1.49, 95% CI 0.86–2.55, *P* = 0.15). The educational level also influenced the infection rate. Compared with the lower infection rate in the higher education group (23.47%, 46/196), the infection rates were higher in those with intermediate education (high school or vocational training, OR 1.11, 95% CI 0.58–2.05, *P* = 0.75) and lower education (secondary education and below, OR 1.38, 95% CI 0.67–2.77, *P* = 0.37). The infection rate among married individuals (OR 1.07, 95% CI 0.51–2.39, *P* = 0.87) was slightly higher than that among unmarried individuals.

**Table 3:** Univariate and multivariate logistic regression analysis of *H. pylori* infection.

Variables	Case/Total (%)	Univariate Logistic Analysis		Multivariate Logistic Analysis	
		OR (95%CI)	*P* value	OR (95%CI)	P-value
Demographic characteristics					
Gender					
Male	28/131 (21.37)	1.00 [Reference]		1.00 [Reference]	
Female	50/183 (27.32)	1.38 (0.82, 2.37)	0.23	1.32 (0.77,2.31)	0.32
Age					
< 30	6/30 (20.00)	1.00 [Reference]		1.00 [Reference]	
30-39	20/70 (28.57)	1.60 (0.59, 4.84)	0.37	1.66 (0.45,6.49)	0.45
40-49	23/85 (27.06)	1.48 (0.56, 4.41)	0.45	1.45 (0.38,5.85)	0.59
50-60	29/129 (22.48)	1.16 (0.46, 3.37)	0.77	1.06 (0.28,4.28)	0.93
Education					
Higher education	46/196 (23.47)	1.00 [Reference]		1.00 [Reference]	
Intermediate: high school or vocational training	18/71 (25.35)	1.11 (0.58,2.05)	0.75	1.17 (0.58,2.32)	0.65
Lower secondary education and below	14/47 (29.79)	1.38 (0.67,2.77)	0.37	1.51 (0.69,3.22)	0.29
Marital status					
Unmarried	10/42 (23.80)	1.00 [Reference]		1.00 [Reference]	
Married	68/272 (25.00)	1.07 (0.51,2.39)	0.87	0.88 (0.32,2.62)	0.81
BMI (Kg/m^2^)					
Normal weight	40/177 (22.60)	1.00 [Reference]			
Underweight	2/11 (18.18)	0.76 (0.11, 3.10)	0.73		
Overweight	33/109 (30.28)	1.49 (0.86, 2.55)	0.15		
Obese	3/17 (17.65)	0.73 (0.16, 2.39)	0.64		
Lifestyle					
Separate dining					
No	37/166 (22.29)	1.00 [Reference]			
Yes	41/148 (27.70)	1.34 (0.80,2.24)	0.27		
Tea drinking					
No	43/198 (21.72)	1.00 [Reference]			
Yes	35/116 (30.17)	1.56 (0.92,2.62)	0.10		
Smoking					
No	68/284 (23.94)	1.00 [Reference]			
Yes	10/30 (33.33)	1.59 (0.68,3.49)	0.26		
Dietary preferences					
Light	9/48 (18.75)	1.00 [Reference]			
Moderate	61/234 (26.07)	1.53 (0.73,3.53)	0.29		
Heavy	8/32 (25.00)	1.44 (0.48,4.29)	0.50		
Alcohol consumption					
No	61/246 (24.80)	1.00 [Reference]			
Yes	17/68 (25.00)	1.01 (0.53,1.85)	0.97		
Sharing water cups					
No	69/279 (24.73)	1.00 [Reference]			
Yes	9/35 (25.71)	1.05 (0.45,2.28)	0.90		

In addition, we analyzed lifestyle habits such as separate dining, tea drinking, smoking, dietary preferences, alcohol consumption, and water cups. Although no significant association was found between these factors and *H. pylori*, we noticed that the infection rate of *H. pylori* was higher among people who practiced separate dining (OR 1.34, 95% CI 0.80–2.24, *P* = 0.27), tea drinking (OR 1.56, 95% CI 0.92–2.62, *P* = 0.10), and smoking (OR 1.59, 95% CI 0.68–3.49, *P* = 0.26). We included gender, age, education level, and marital status in the multivariate analysis to further assess their relationship with *H. pylori* infection. However, this study did not identify any risk factors significantly associated with *H. pylori* infection (see **Table 3**).

## Discussion

This study aims to assess the prevalence, antibiotic resistance, and associated risk factors of *H. pylori* infection in the urban population of Dongying City, China. Non-invasive methods for detecting *H. pylori* infection status and antibiotic resistance are crucial. The study by Losurdo et al. demonstrated that non-invasive molecular detection in fecal samples is a feasible and effective method for accurately detecting *H. pylori* infection and its antibiotic resistance (Losurdo et al., 2020). However, considering the potential interference caused by the microbial diversity in fecal samples, we used the string test combined with qPCR to determine *H. pylori* infection status and antibiotic resistance in gastric fluid samples (Wang et al., 2024). The string test method effectively reduces the invasiveness of obtaining gastric mucosal tissue and fluid samples through endoscopy. Furthermore, using gastric fluid samples helps avoid sampling representativeness issues caused by localized *H. pylori* colonization in the gastric mucosa (Yang et al., 2023). The combination of qPCR enables precise detection of multiple targets by selecting different primers and probes, allowing for the simultaneous detection of *H. pylori* infection status and its antibiotic resistance (Pabinger et al., 2014).

The prevalence of *H. pylori* infection shows a declining trend. Li et al. reported that the global estimated prevalence of *H. pylori* decreased from 58.2% (95%CI: 50.7–65.8) between 1980 and 1990 to 43.1% (95%CI: 40.3–45.9) between 2011 and 2022 (Xie et al., 2024). Our results show that the infection rate of *H. pylori* in Dongying City, Shandong Province, is 24.84%, which is lower than the overall infection rate of 36.7% diagnosed by Kong et al. using the ^13^C-urea breath test on 1,173 participants in six regions of Shandong Province (Kong et al., 2022). The prevalence of *H. pylori* infection varies widely among countries and regions (Xu et al., 2014). Our study found a lower infection rate than the prevalence rates obtained using the same detection methods, such as 27.08% in urban areas of China (Wang et al., 2024). Specifically, when compared to other Chinese cities, such as Quanzhou, which reported a rate of 52.60% (Xie et al., 2025), the infection rate observed in our study was significantly lower.

Appropriate antibiotic selection and combination therapy are key to successfully eradicating *H. pylori*. Currently, guidelines in China recommend *H. pylori* eradication with bismuth quadruple therapy (BQT) and high-dose dual therapy (HDDT) (Ding et al., 2022). Bismuth quadruple therapy includes a proton pump inhibitor, bismuth, metronidazole, and either tetracycline or amoxicillin. High-dose dual therapy consists of high doses of a proton pump inhibitor and amoxicillin. In our study, *H. pylori*-positive patients from Dongying City in Shandong Province showed resistance rates to clarithromycin and levofloxacin above the 15% threshold. Existing data show that in most provinces, the primary and secondary resistance rates to clarithromycin, metronidazole, and levofloxacin exceed 15%, with clarithromycin at 30.72% (95% CI: 27.53%–33.99%), metronidazole at 70.14% (95% CI: 29.53%–37.46%), and levofloxacin at 32.98% (95% CI: 28.73%–37.37%). However, the resistance rates to amoxicillin, tetracycline, and furazolidone are generally below 15%, with amoxicillin at 2.41% (95% CI: 1.43%–3.60%), tetracycline at 2.53% (95% CI: 1.19%–4.28%), and furazolidone at 1.54% (95% CI: 0.28%–3.62%) (Zeng et al., 2024). Notably, resistance varies significantly across different regions. The clarithromycin resistance rate is relatively low in the eastern (24%) and southern regions (24%), but higher in the western regions. Similarly, the resistance to metronidazole has decreased in the northern regions, dropping from 71% to 62% over the years, but remains high in other areas (Chen et al., 2022). These regional differences may be related to various factors such as local antibiotic usage habits, distribution of healthcare resources, patient adherence to treatment, and regional microbial community variations.

Factors associated with *H. pylori* infection have not been established (Yan et al., 2013). Regional factors such as diet, sanitation, and healthcare access significantly influence *H. pylori* infection rates and resistance patterns. Although this study analyzed several risk factors for *H. pylori* infection, excluding socioeconomic status and family history may lead to confounding bias. According to previous studies (Zhou et al., 2023), these factors significantly impact *H. pylori* infection. Therefore, future studies should incorporate these factors to more comprehensively assess the risks. However, we could not identify any independent risk factors for *H. pylori* infection in this study. This result may be related to the study design, sample characteristics, or other uncontrolled variables and warrants further investigation. Our findings provide important insights into the prevalence of antibiotic resistance among *H. pylori* strains, particularly for commonly used antibiotics such as clarithromycin and levofloxacin. These data are essential for clinicians in tailoring treatment strategies that are both effective and aligned with local resistance patterns.

This study’s strength lies in using highly accurate methods to detect *H. pylori*. However, it has limitations. First, the cross-sectional design prevents establishing causal links between *H. pylori* and risk factors. Second, random enrollment without stratified sampling may limit population representativeness. Although Dongying’s residents come from diverse regions, the small sample size restricts generalizability. Third, this study did not conduct a detailed analysis of risk factors related to antibiotic resistance, such as demographic factors and previous antibiotic use. This limitation may affect the interpretation of the study results. Future research should further explore the impact of these factors on antibiotic resistance rates to guide targeted resistance mitigation strategies. Finally, the lack of methodological comparisons within this cohort may affect result reliability, and the method’s applicability to other populations requires further validation. Future research should aim to expand its scope, focusing on a comprehensive analysis of *H. pylori* resistance patterns. As commonly used antibiotics in *H. pylori* eradication regimens, metronidazole and amoxicillin resistance patterns merit in-depth investigation. Previous studies have reported that the resistance rate of metronidazole in China exceeds 50%, while the resistance rate of amoxicillin remains relatively low. Future studies should also consider increasing sample sizes and optimizing study designs, such as incorporating longitudinal studies to establish causal relationships and employing stratified sampling methods to enhance population representativeness. These efforts would further improve the findings’ scientific rigor and practical applicability.

## Conclusion

Among the urban population on the eastern coast of Dongying, China, the infection rate of *H. pylori* was 24.84%, with clarithromycin resistance at 60.25% and levofloxacin resistance at 46.15%. Females are more susceptible to *H. pylori* infection than men. These findings will aid in formulating specific measures to prevent *H. pylori* infection in Dongying Province. At the same time, the updated resistance rates to Clarithromycin and Levofloxacin provide local guidance for the personalized clinical treatment of bacterial infections. This approach will help reduce antibiotic resistance and improve eradication success rates, ultimately lowering the overall prevalence of *H. pylori* infection in the region.

## Data Availability

The raw data supporting the conclusions of this article will be made available by the authors, without undue reservation.
